# Patterns of paediatric end-of-life care: a chart review across different care settings in Switzerland

**DOI:** 10.1186/s12887-018-1021-2

**Published:** 2018-02-16

**Authors:** Karin Zimmermann, Eva Cignacco, Sandra Engberg, Anne-Sylvie Ramelet, Nicolas von der Weid, Katri Eskola, Eva Bergstraesser, on behalf of the PELICAN Consortium, Marc Ansari, Christoph Aebi, Reta Baer, Maja Beck Popovic, Vera Bernet, Pierluigi Brazzola, Hans Ulrich Bucher, Regula Buder, Sandra Cagnazzo, Barbara Dinten, Anouk Dorsaz, Franz Elmer, Raquel Enriquez, Patricia Fahrni-Nater, Gabi Finkbeiner, Bernhard Frey, Urs Frey, Jeannette Greiner, Ralph-Ingo Hassink, Simone Keller, Oliver Kretschmar, Judith Kroell, Bernard Laubscher, Kurt Leibundgut, Reta Malaer, Andreas Meyer, Christoph Stuessi, Mathias Nelle, Thomas Neuhaus, Felix Niggli, Geneviève Perrenoud, Jean-Pierre Pfammatter, Barbara Plecko, Debora Rupf, Felix Sennhauser, Caroline Stade, Maja Steinlin, Lilian Stoffel, Karin Thomas, Christian Vonarburg, Rodo von Vigier, Bendicht Wagner, Judith Wieland, Birgit Wernz

**Affiliations:** 1 Department Public Health (DPH), Nursing Science, University of Basel, Bernoullistrasse 28, 4056 Basel, Switzerland; 2 Paediatric Palliative Care, University Children’s Hospital Zurich, Children’s Research Center CRC, Steinwiesstrasse 75, 8032 Zurich, Switzerland; 3 Department of Pediatrics, Inselspital Bern University Hospital, Bern, Switzerland; 4 Health Division, University of Applied Sciences Bern, Bern, Switzerland; 5 School of Nursing, University of Pittsburgh, 3500 Victoria Street, Pittsburgh, PA 15261 USA; 6 Institute of Higher Education and Research in Healthcare – IUFRS, University of Lausanne, Route de la Corniche 10, 1010 Lausanne, Switzerland; 7 Nurse Research Consultant, Department of Woman-Mother-Child, Lausanne University Hospital CHUV, Lausanne, Switzerland; 8 Paediatric Haematology-Oncology, University Children’s Hospital UKBB, Spitalstrasse 33, 4056 Basel, Switzerland; 9 Triemli Hospital Zurich, Zurich, Switzerland

**Keywords:** End-of-life care, Terminal care, Paediatrics, Neonatology, Child, Practice patterns, Retrospective studies

## Abstract

**Background:**

Paediatric **e**nd-of-life care is challenging and requires a high level of professional expertise. It is important that healthcare teams have a thorough understanding of paediatric subspecialties and related knowledge of disease-specific aspects of paediatric end-of-life care. The aim of this study was to comprehensively describe, explore and compare current practices in paediatric end-of-life care in four distinct diagnostic groups across healthcare settings including all relevant levels of healthcare providers in Switzerland.

**Methods:**

In this nationwide retrospective chart review study, data from paediatric patients who died in the years 2011 or 2012 due to a cardiac, neurological or oncological condition, or during the neonatal period were collected in 13 hospitals, two long-term institutions and 10 community-based healthcare service providers throughout Switzerland.

**Results:**

Ninety-three (62%) of the 149 reviewed patients died in intensive care units, 78 (84%) of them following withdrawal of life-sustaining treatment. Reliance on invasive medical interventions was prevalent, and the use of medication was high, with a median count of 12 different drugs during the last week of life. Patients experienced an average number of 6.42 symptoms. The prevalence of various types of symptoms differed significantly among the four diagnostic groups. Overall, our study patients stayed in the hospital for a median of six days during their last four weeks of life. Seventy-two patients (48%) stayed at home for at least one day and only half of those received community-based healthcare.

**Conclusions:**

The study provides a wide-ranging overview of current end-of-life care practices in a real-life setting of different healthcare providers. The inclusion of patients with all major diagnoses leading to disease- and prematurity-related childhood deaths, as well as comparisons across the diagnostic groups, provides additional insight and understanding for healthcare professionals. The provision of specialised palliative and end-of-life care services in Switzerland, including the capacity of community healthcare services, need to be expanded to meet the specific needs of seriously ill children and their families.

## Background

Despite continued advancements in medical care and improved (expected) survival, infant and childhood deaths due to complex chronic conditions (CCC) or prematurity are inevitable [1]. Deaths during the first year of life constitute approximately 50% of disease-related infant and childhood deaths in developed countries, the causes of which include perinatal complications, prematurity, or congenital anomalies [2, 3]. Beyond the age of one year, the three most common life-limiting CCCs are neurological/neuromuscular and cardiovascular conditions (including genetic disorders), and malignancies [4, 5]. The majority of disease- and prematurity-related deaths occur in hospitals, [6, 7], and for children dying at home, hospital use in their terminal stage is high [1, 4]. Symptom burden and reliance on medical technology has been reported to be considerable [3, 8]. Circumstances and characteristics of deaths, however, are known to vary by age and medical conditions [1, 4].

Paediatric palliative care (PPC) emerged as a medical subspecialty aimed at meeting the specific needs of seriously ill children and their families. According to the World Health Organization (WHO) “palliative care is an approach that improves the quality of life of patients and their families facing the problem associated with life-threatening illness, through the prevention and relief of suffering by means of early identification and impeccable assessment and treatment of pain and other problems, physical, psychosocial and spiritual” [9]. More specifically and as part of palliative care, the term end-of-life (EOL) care refers to care when death is imminent [10]. Meeting the needs of affected children and their families requires a wide-ranging and integrative approach from a compassionate and skilled multidisciplinary team [11]. PPC and EOL care should be provided in all settings where it is required [12]; although, specialised PPC teams are mostly hospital based [13]. A thorough understanding of paediatric subspecialties and related knowledge of disease-specific aspects of paediatric EOL are needed. This understanding should go beyond the horizon of a single hospital and take into account the heterogeneous settings where care can be provided (tertiary settings, general hospitals, paediatric primary care and in the community). There is not much evidence on which to base best practice, and most existing studies focus on specific diagnostic groups or specific care settings [1, 14, 15]. It was therefore the aim of this national study to comprehensively describe, explore and compare current practices in paediatric EOL care in four distinct diagnostic groups (cardiology, neonatology, neurology and oncology) across healthcare settings including all relevant levels of healthcare providers in Switzerland.

## Methods

### Study design

This retrospective chart review was part of PELICAN (Paediatric End-of-LIfe-CAre Needs in Switzerland), a nationwide study “to provide comprehensive information and to understand the current practice of EOL care (i.e. in this study and similar to other studies, the last 4 weeks of life prior to death [16]) in paediatric settings in Switzerland (hospital and community care) and to explore and describe parental perspectives and the perspectives of the healthcare professionals involved” [17]. Human Research Ethics Committees from the 11 Swiss cantons in which the study took place approved the PELICAN study. Parents who had lost a child due to a cardiac, neurological or oncological condition or during the neonatal period (independent of the underlying condition) in the years 2011 and 2012 were invited to participate. Neonates < 24 h of life and patients > 18 years were excluded. Information on how, where and when recruitment took place is described in detail elsewhere [18].

### Setting and data collection

Data from all eligible patients, whose parents had consented to the review of their child’s medical chart, were collected in 13 hospitals, 2 institutions and 10 community-based healthcare service providers throughout Switzerland. Among the 13 hospitals, there were 5 tertiary paediatric centres, 4 dedicated children’s hospitals, 3 general hospitals with paediatric units and 1 tertiary care centre with a neonatal intensive care unit. A multiprofessional PPC team was available in two tertiary paediatric centres and one dedicated children’s hospital; no paediatric hospices exist in Switzerland.

Data collection was conducted mainly by the first author, who also developed the coding manual and all case report forms as well as instructing and supervising five assistants, who supported data collection [19]. The coding manual was developed within the PELICAN study group [17] and pilot tested with 10 children who were treated in 5 different hospitals and died in the year 2010. In accordance with this study’s definition of EOL care as care during the last four weeks of life, data collection was restricted to the 28 days prior to the child’s death. All extracted data was entered into secuTrial®, a browser-based electronic data capture system (InterActive Systems, Berlin, Germany). During the first two months of data collection, 5 % of the medical records reviewed by one of the five assistants were randomly selected and audited by KZ by performing a dual review. Any data entry discrepancies were checked for its nature of assessment error. No systemic data entry errors were detected. There were very few data entry discrepancies and those that occurred were almost always related to mixed documentation quality in the medical records that left room for interpretation, e.g., change of do-not-resuscitate (DNR) order. Emerging questions around those inconsistencies were continuously discussed among data collectors. Variable instructions in the manual were revised as needed to ensure the quality of ongoing data extraction and reduce the likelihood of inter-rater discrepancies [19].

### Variables

The following data were collected for this study: (1) demographics (age, gender); (2) diagnostic information (the underlying diagnosis primarily responsible for the patient’s death, gestational age for newborns only, time since diagnosis, and whether the diagnosis was made prenatally); (3) circumstances of death (place of death, occurrence of resuscitation, existence of DNR orders and whether these orders changed during the last four weeks of life, and treatment withdrawal); (4) interventions (at least once during the last four weeks of life, Yes-No: anaesthesia, e.g., surgery, imaging; ventilation; central access device; enteral feeds) and medications (number and types of medications were recorded only for the last two weeks of life to reduce the time burden related to reviewing the medical records); (5) symptoms (presence of various symptoms); (6) hospital and community healthcare utilisation (hospital days and admissions, days spent at home, number of days and hours, and types of care provided by community services). We also assessed whether the treatment approach was documented as palliative care and whether this approach changed during the last four weeks of life.

A diagnostic chapter and code from the International Statistical Classification of Diseases and Related Health Problems (ICD), 10th Revision, online version 2016 [20] was assigned to each patient, based on the exact diagnostic information extracted from the patient’s last medical report. Coding was done by two independent appraisers to establish reliability and any discrepancies were discussed until there was consensus about the diagnosis. All symptoms documented in the patient’s chart were recorded during data collection. The ones most frequently reported were grouped into 20 symptoms categories, based on symptoms most frequently reported in the literature [8, 14, 21]. Symptoms that affected similar areas, e.g. spasticity/dystonia for muscular impairments, or agitation/irritability for behavioural problems, were grouped.

### Statistical analysis

Descriptive statistics (measures of central tendency and dispersion, frequencies and percentages) were used to explore and summarize all variables. A binary logistic model with likelihood ratio statistics was utilised for two-tailed comparisons between the diagnostic groups of variables with a binominal response (Yes – No). For count outcome variables, negative binomial regression was utilised to adjust for overdispersion [22]. For variables with a categorical response, equivalence of proportions between diagnostic groups was tested in contingency tables using the Pearson’s chi-square test or Fisher’s exact test when cell sizes were < 5. No measures of missing value replacement were pursued. Due to the multiple comparisons performed, we set a conservative *p*-value of < 0.001 to indicate statistical significance. Statistical analyses were performed using IBM© SPSS© Statistics 21 for Mac® (IBM Corp, Armonk, NY, USA).

## Results

Of the 307 eligible families, 267 could be contacted and were invited to participate in the PELICAN study. Of those, 147 families (55%) consented. Two families lost twins resulting in a study sample of 149 neonates, children and adolescents (Table 1). With neonates comprising 38% of the sample, the median age at death was 0.5 years for the entire sample but substantially higher (*Mdn* = 8.4, *range* = 1.7 -17.4 years) for the oncology group. The neonates' median age was 5 days (*range* = 1 - 26) and substantially lower than age in the other diagnostic groups (Table 1). Seven ICD-10 diagnostic chapters were represented in our four groups’ categorisation, with the highest variety found within the neurology group. The median time between diagnosis of the life-limiting CCC (made after birth) and death for the total sample was one month (*interquartile range [IQR],* 0 – 6). Within the four groups, the median time between diagnosis and death was longest for the neurology group (*Mdn* = 6 months, *IQR* = 3 – 29). Diagnoses made prenatally, were significantly more frequent in the cardiology group compared to the other groups (*p* = < 0.001) and not present in the oncology group. Information related to gestational age was missing for 5 neonates (8.8%) and information related to diagnoses made prenatally for 2 patients (1.3%) (Table 1).

**Table 1:** Demographic and diagnostic patient characteristics

Characteristics	Total *N* = 149 (100%)	Cardiology *n* = 19 (13%)	Neonatology *n* = 57 (38%)	Neurology *n* = 36 (24%)	Oncology *n* = 37 (25%)
Age at death, *Mdn* (*range*)					
in months	6 (0 – 209)	6 (1 – 109)	Na	19 (1 – 207)	101 (20 – 209)
in years	0.5 (0.0 – 17.4)	0.5 (0.1 – 9.1)	Na	1.6 (0.1 – 17.2)	8.4 (1.7 – 17.4)
Gender, *n* (*%*)					
Female	72 (48)	10 (53)	32 (56)	15 (42)	15 (40)
Male	77 (52)	9 (47)	25 (44)	21 (58)	22 (60)
ICD-10 chapter, description, *n* (*%*)					
II Neoplasms	36 (24)	0 (0)	0 (0)	0 (0)	36 (97)
III Blood/immune system	1 (1)	0 (0)	0 (0)	0 (0)	1 (3)^a^
IV Endocrine, nutritional, metabolic	6 (4)	0 (0)	0 (0)	6 (16)	0 (0)
VI Nervous system	21 (14)	0 (0)	2 (4)	19 (53)	0 (0)
IX Circulatory system^b^	5 (3)	4 (21)	0 (0)	1 (3)	0 (0)
XVI Conditions originating in perinatal period	45 (30)	0 (0)	44 (77)	1 (3)	0 (0)
XVII Congenital, chromosomal	35 (24)	15 (79)	11 (19)	9 (25)	0 (0)
Gestational age (for the neonatology group only)			*n* = 52^c^		
24 0/7 – 27 6/7	Na	Na	17 (33)	Na	Na
28 0/7 – 31 6/7	Na	Na	8 (15)	Na	Na
32 0/7 – 36 6/7	Na	Na	9 (17)	Na	Na
37 0/7 - > 42 0/7	Na	Na	18 (35)	Na	Na
Time since diagnosis^d^					
in days, *Mdn* (*range*)	Na	Na	4 (1 – 26)	Na	Na
in months, *Mdn* (*range*)	1 (0 – 205)	7 (0 - 66)	Na	9 (0 - 205)	4 (0 - 139)
in years, *Mdn* (*range*)	0 (0 – 17)	0.5 (0.5 – 5.5)	Na	0.5 (0.0 – 17.0)	0.5 (0.0 – 12.0)
Diagnosis made prenatally	*n* = 147^c^			*n* = 34^c^	
Yes, *n* (*%*)	31 (21)	11 (58)	13 (23)	7 (21)	0 (0)

*Na* Not applicable, *ICD-10* International Classification of Diseases, 10th Revision

^a^Aplastic anaemia

^b^Stroke included

^c^Information was missing for some cases

^d^Calculated from date of birth, even if diagnosis was suspected prenatally

### Place and circumstances of death

Ninety-three patients (62%) died in an intensive care unit (ICU), with the highest proportion of ICU deaths occurring in the neonatology group (Table 2). Twenty-five patients (17%) died at home, with the highest proportion of home deaths occurring in the oncology group. Twenty-six patients (17%) received cardiopulmonary resuscitation (CPR) within 24 h before death, despite 15 patients (17%, *n* = 147) had a documented DNR order. A DNR order was documented in 91 patients’ charts (62%). Of those, 51 patients (57%) had a change of the DNR order within the last four weeks of life. This change occurred most frequently in the neonatology group (Table 2), often within hours before the child’s death. For 78 patients (84%) of the 93 who died in an ICU, death was preceded by a decision to withdraw life-sustaining interventions.

**Table 2:** Place and circumstances of death

	Total *N* = 149 (100%)	Cardiology *n* = 19 (13%)	Neonatology *n* = 57 (38%)	Neurology *n* = 36 (24%)	Oncology *n* = 37 (25%)	*p*-value
Place of death, *n* (*%*)						< 0.001^a^
PICU	63 (42)	13 (67)	27 (48)	13 (36)	10 (27)	
NICU	30 (20)	0 (0)	27 (48)	3 (8)	0 (0)	
Hospital ward / long-term institution	26 (18)	2 (11)	0 (0)	13 (36)	11 (30)	
Home	25 (17)	2 (11)	2 (3)	7 (20)	14 (38)	
Emergency department / Transport	5 (3)	2 (11)	1 (1)	0 (0)	2 (5)	
CPR^b^						
Yes, *n* (*%*)	26 (17)	7 (37)	6 (11)	7 (19)	6 (16)	0.097^c^
DNR order						
Yes, *n* (*%*)	91 (62)	11 (58)	20 (35)	33 (92)	27 (77)	< 0.001^c^
DNR order change within the last four weeks of life						
Yes, *n* (*%*)	51 (57)	7 (64)	18 (90)	13 (41)	13 (48)	0.002^c^
Withdrawal of life-sustaining interventions^d^	*n* = 93	*n* = 13	*n* = 54	*n* = 16	*n* = 10	
Yes, *n* (*%*)	78 (84)	10 (77)	49 (91)	12 (75)	7 (70)	0.203^c^

*PICU* Paediatric intensive care unit, *NICU* Neonatal intensive care unit, *CPR* Cardiopulmonary resuscitation, *DNR* Do not resuscitate

^a^Across the four groups, based on Fisher’s exact test

^b^Within 24 h before death

^c^Across the four groups, based on likelihood ratio chi-square

^d^Only applies to patients who died in an intensive care unit

### Interventions, medication and symptoms

**Fig. 1:**
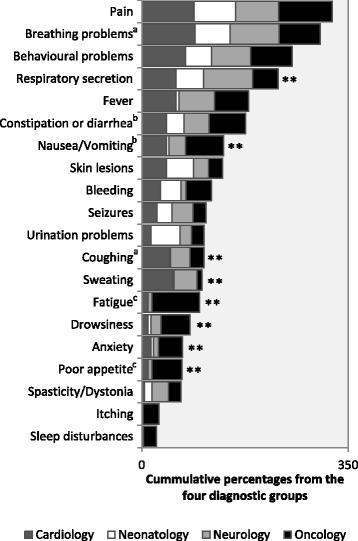
Symptom prevalence and comparison between the four diagnostic groups. ** = *p*-value < 0.001 based on a negative binomial regression model.^a^Adjusted for mechanical ventilation. ^b^Adjusted for enteral feeds. ^c^Neonatology group excluded due to 0% of symptom presence

Patients underwent several interventions, suffered from a variety of symptoms, and received a considerable amount of medication, as documented in their charts. This information is detailed in Table 3 and Fig. 1. Fifty-one patients (34%) received anaesthesia at least once during their last four weeks of life, some patients more than once and for different interventions. The most commonly documented interventions requiring anaesthesia were surgical interventions in 28 patients (55% of the 51 patients who received anaesthesia) and diagnostic procedures, e.g. imaging in 27 patients (53%). The overall median and mean number of medications with orders for standard daily doses and as-needed orders rose from 9 (*range* = 0 - 42), 12 (*SD* = 9.20) respectively during the second-to-last week to 12 (*range* = 1 – 46), 14 (*SD* = 9.15) respectively during the last week. For 133 patients (89%) the last treatment approach was documented as palliative. The approach changed from curative to palliative during the last month in 88 patients (59%), most commonly in the neonatology group and least commonly in the oncology group (90% vs. 32%, *p* = < 0.001). Information was missing for six variables related to interventions and medication and ranged between 0.7% and 2.7% (Table 3).

**Table 3:** Interventions and medications during the last four weeks of life

	Total *N* = 149 (100%)	Cardiology *n* = 19 (13%)	Neonatology *n* = 57 (38%)	Neurology *n* = 36 (24%)	Oncology *n* = 37 (25%)	*p*-value
Interventions requiring anaesthesia	*n* = 148^a^			*n* = 35^a^		
Yes, *n* (*%*)	51 (34)	11 (58)	21 (37)	6 (17)	13 (35)	0.021^b^
Mechanical ventilation						
Yes, *n* (*%*)	94 (63)	14 (74)	55 (97)	15 (42)	10 (27)	< 0.001^b^
ECMO						
Yes, *n* (*%*)	7 (5)	4 (21)	3 (5)	0 (0)	0 (0)	< 0.001^b^
Presence of CAD	*n* = 148^a^				*n* = 36^a^	
Yes, *n* (*%*)	106 (72)	14 (74)	55 (97)	12 (33)	25 (69)	< 0.001^b^
Enteral feeds via nasogastric or gastrostomy tube	*n* = 148^a^			*n* = 35^a^		
Yes, *n* (*%*)	114 (77)	17 (90)	51 (90)	33 (94)	13 (35)	< 0.001^b^
Medication count in the last week of life^c^	*n* = 146^a^			*n* = 35^a^	*n* = 35^a^	
*Mdn, (range)*	12 (1 - 46)	19 (3 - 45)	12 (1 - 34)	10 (3 - 39)	13 (4 - 46)	0.006^b^
*Mean, (SD)*	14 (9.15)	21 (11.73)	12 (7.02)	13 (8.82)	15 (9.69)	
Pain medication	*n* = 148^a^			*n* = 35^a^		
Yes, *n* (*%*)	140 (95)	18 (95)	54 (95)	33 (94)	35 (95)	1.000^b^
Anxiolytic medication	*n* = 145^a^			*n* = 35^a^	*n* = 34^a^	
Yes, *n* (*%*)	84 (58)	14 (74)	27 (47)	18 (51)	25 (74)	0.032^b^
Antiemetic medication	*n* = 146^a^			*n* = 35^a^	*n* = 35^a^	
Yes, *n* (*%*)	25 (17)	2 (11)	0 (0)	0 (0)	23 (66)	< 0.001^b^

*ECMO* Extracorporeal membrane oxygenation, *CAD* Central access device, either venous or arterial

^a^Information was missing for some cases

^b^Across the four groups, based on likelihood ratio chi-square

^c^Includes both standing daily dosages and as-needed orders

Pain was the most frequently documented symptom, and occurred in 110 patients (78%, *N* = 141), with no significant differences between the diagnostic groups. One hundred and forty patients (95%, *N* = 148) received some pain medication, most commonly opioids (93%), followed by paracetamol (67%). Other common symptoms included breathing problems (*n* = 107, 72%), followed by behavioural problems such as agitation or irritability (*n* = 89, 60%). Some symptoms, such as respiratory secretion, fever, nausea/vomiting, coughing, sweating, fatigue, drowsiness, anxiety (including worry and sadness), and poor appetite, differed significantly (*p* = < 0.001) between the diagnostic groups (Figure 1). Overall, an average of 6.42 (*SD* = 3.14) symptoms were recorded per patient. Significantly fewer symptoms were reported in neonates (*M* = 4.39, *SD* = 2.15) compared to all other groups (*p* = < 0.001).

### Hospital and community healthcare utilisation

Overall, our study patients stayed in the hospital for a median of six days (*IQR* = 2 – 19) during their last four weeks of life, with the highest number of hospital days for patients in the cardiology group (Table 4). Nineteen patients (13%) had no hospital days: 11 of them (58%) from the oncology group, 5 (26%) from the neurology group, 3 (16%) from the cardiology group, and none from the neonatology group. Among the 130 patients who had at least one hospital day, 62 patients (48%) had one hospital admission, 10 patients (8%), and 2 patients (1%) had 3 admissions during the last four weeks of life. Fifty-six patients (43%) had zero hospital admissions, meaning that those patients were hospitalised at the beginning of data collection and remained there until their death or discharge. Of the 57 patients in the neonatology group, 23 patients (40%) were born in a hospital with no ICU and had to be transferred to a referral tertiary hospital with an ICU. Patients from the other diagnostic groups were most commonly admitted from home (Table 4).

**Table 4:** Hospital and community healthcare utilisation during the last four weeks of life

	Total *N* = 149 (100%)	Cardiology *n* = 19 (13%)	Neonatology *n* = 57 (38%)	Neurology *n* = 36 (24%)	Oncology *n* = 37 (25%)	*p*-value
Hospital days, *Mdn* (*range*)	6 (0 - 28)	20 (0 - 28)	5 (1 - 26)	7 (0 - 28)	4 (0 - 28)	0.035^a^
Care setting before hospital admission^b^, *n* (*%*)	*n* = 88	*n* = 17	*n* = 23	*n* = 26	*n* = 22	
Home	37 (42)	11 (64)	0 (0)	16 (62)	10 (45)	NA^c^
Other hospital	35 (40)	3 (18)	23 (100)	4 (15)	5 (23)	NA^c^
Emergency department	8 (9)	3 (18)	0 (0)	4 (15)	1 (5)	NA^c^
Outpatient clinic	7 (8)	0 (0)	0 (0)	1 (4)	6 (27)	NA^c^
Long-term institution	1 (1)	0 (0)	0 (0)	1 (4)	0 (0)	NA^c^
Patients at home at least for one day, *n* (*%*)	72 (48)	11 (58)	3 (5)	27 (75)	31 (84)	< 0.001^a^
Days spent at home, *Mdn* (*range*)	0 (0 - 28)	8 (0 - 28)	0 (0 - 16)	21 (0 - 28)	24 (0 - 28)	0.001^a^
Care days with community care service^d^,	*n* = 72	*n* = 11	*n* = 3	*n* = 27	*n* = 31	
*Mdn* (*range*)	1 (0 - 28)	0 (0 - 24)	1 (0 - 5)	5 (0 - 28)	0 (0 - 28)	0.001^a^
Hours of care by community care service^d^	*n* = 36	*n* = 5	*n* = 2	*n* = 15	*n* = 14	
*Mdn* (*range*)	34 (2 - 315)	12 (7 - 190)	6 (3 - 8)	38 (4 - 315)	23 (2 - 108)	0.111^a^
Type of community care service						
Family education/support, *n* (*%*)	35 (97)	5 (100)	2 (100)	14 (93)	14 (100)	NA^c^
Needs assessment, *n* (*%*)	31 (86)	4 (80)	1 (50)	13 (87)	13 (93)	NA^c^
Monitoring of vital signs/general condition, *n* (*%*)	29 (81)	5 (100)	2 (100)	13 (87)	9 (64)	NA^c^
Administration of medication, *n* (*%*)	25 (69)	5 (100)	0 (0)	10 (67)	10 (71)	NA^c^
Interventions related to enteral feeds, *n* (*%*)	22 (61)	5 (100)	1 (50)	13 (87)	3 (21)	NA^c^
Respiratory interventions, *n* (*%*)	18 (50)	2 (40)	0 (0)	11 (73)	5 (36)	NA^c^
Interventions related to excretion, *n* (*%*)	14 (39	1 (20)	0 (0)	6 (40)	7 (50)	NA^c^

*NA* Not applicable

^a^Across the four groups, based on likelihood ratio chi-square

^b^Representing the cumulative hospital admissions in all patients

^c^No significance testing conducted due to small numbers

^d^Consisting of nurses, most of them specialised in paediatric and/or community nursing

Seventy-two patients (48%) stayed at home for at least one day, with patients from the oncology group having the highest number of home days (*Mdn* = 24, *IQR* = 4 - 28), followed by patients from the neurology group (*Mdn* = 21, *IQR* = 4 - 26). Of the 72 patients who stayed at home, 36 (50%) received professional care from a community-based service. The provision of education and support to empower the family was the most commonly provided service, as documented by the care provider, and patients from the neurology group received more care hours than patients from the other groups (Table 4).

## Discussion

There are several principal findings in this nationwide study examining patterns of care at EOL in four distinct diagnostic groups: patients had a variety of primary diagnoses, covering seven different ICD-10 diagnostic chapters; 62% of all patients died in ICUs, with 84% of them following a decision to withdraw life-sustaining treatment; reliance on invasive medical interventions was prevalent and patients were exposed to multiple medications; patients experienced many symptoms with an average of six symptoms per patient; finally, community-based health care services were involved in only half of the cases of the 72 patients (48%) of patients who spent time at home during their last four weeks of life.

### Strengths and limitations

The study provides a wide-ranging overview of current EOL care practices in a heterogeneous real-life setting of hospitals, long-term institutions and community healthcare organisations. The inclusion of patients with all major diagnoses leading to disease- and prematurity-related infant and childhood deaths, as well as comparisons across the diagnostic groups, provides additional insight and understanding for healthcare professionals. Previous studies in this field have frequently been limited to the hospital setting [1, 3] or to a specific diagnostic group [8, 14, 15]. Our study is limited by its cross-sectional, primarily descriptive design incorporating a retrospective chart review. This approach does not allow conclusions to be drawn about care quality and quality of life at the EOL. Generalisability is further limited by the way that EOL care is often delivered in Switzerland. Specialised PC/EOL care was offered in only 3 of the 13 hospitals where data were collected, and no children’s hospices exist in Switzerland. The study’s definition of EOL care as the last four weeks of life is somehow artificial, yet was justified by the literature, experts’ opinion and feasibility constraints. The study, therefore, provides insight in paediatric EOL care in a limited time frame, not necessarily covering all aspects of EOL care across a broader time period. Known reliability issues related to chart reviews were kept to a minimum by utilising established and appropriate measures, resulting in few discrepancies in the data collected. However, the mixed quality of documentation among healthcare personnel, resulting in incomplete or missing data independent of data collection quality still limits the study’s reliability [19]. The comparisons between the four major diagnostic groups highlight elements that warrant discussion.

### Medication and symptoms

Medication counts in our study were high, with an overall mean of 14 drugs or as-needed medication orders during the last week of life. This number is higher than the reported average of 9 drugs in a study involving 515 paediatric patients with a similar diagnostic profile receiving PPC [3]. A similar average number of 13.9 (*SD* = 8.9) of medications used in the last week of life was reported in a study of 30 children dying in an Australian hospital in 2001. Two thirds of the 30 children died in the ICU, which might have influenced the high numbers found in this study [23]. We found that the medication count increased from the second-to-last week to the last week of life. Thus, it seems that the intensity of medical treatment increases as the child nears death, a phase which is accompanied by a greater need for pharmacological interventions, especially for relieving pain, based on our clinical experience. The high number of medications in the cardiology group was often due to the frequent need for CPR and a high prevalence of surgical interventions, which are also described in other studies with cardiology patients [15, 21]. Although not perfectly comparable, symptom type and prevalence differed from the aforementioned study of 515 patients receiving PPC, in which pain was only the sixth most frequent symptom extracted from patients’ charts [3]. However, pain has been reported to be the most frequent symptom in other studies with various paediatric cohorts in PC or EOL care [8, 14]. Our study adds to existing knowledge by demonstrating that symptom prevalence is dependent on the underlying CCC and that it can differ considerably.

### Hospital and community healthcare utilisation

Most patients (*n* = 130) stayed at least one day in hospital during their last four weeks of life. Of those, 62 patients (48%) had at least one hospital admission and 10 patients (8%) had more than one admission. This is a lower percentage than reported in a recent North American study about trends in high-intensity EOL care among children with cancer [24]. In this population-based study with a cohort of 815 children diagnosed with cancer who died between 2000 and 2012, 143 patients (17.6%) of the patients had more than one hospital admission within 30 days of death. Compared with our oncology sub-group (*n* = 37), twenty-six patients stayed at least one day in hospital and 3 (11%) of them had more than one admission during their last four weeks of life.

Slightly less than 50% of our study’s patients were at home at some point during their last four weeks of life. Naturally, this was the case for very few neonates. In light of the probably growing rate of prenatal diagnosis of a life-limiting CCC, early initiation of PPC may allow better planning and implementation of specialised care services at home [25]. Perinatal palliative care is an even younger specialty than PPC [25] and was not integrated in the three PPC programmes in Switzerland. A recent survey about the provision of services showed that perinatal palliative care programmes in the US were based in academic medical centres, regional and local hospitals, or community based hospitals [26]. Back in 2013, Feudtner et al. [13] showed however, that only 54% of hospital-based PPC programmes provided prenatal consultation [13].

Only half of the patients who spent time at home received community-based healthcare services. Recent data from Germany and the US show that the coordination and provision of specialised palliative home care can alleviate caregivers’ distress and burden [27], and improve both the child’s [28] and the caregivers’ quality of life [27]. As reported by our study, community nursing care encompasses a range of service types. The high level of coordination with the leading team in the hospital and the expertise required makes it especially challenging. Subgroup analysis of our study’s at-home population targeting facilitators for and barriers to EOL care in the home setting has been performed and is published elsewhere [29].

### Implications

PPC is growing internationally and the provision of consultation by a hospital-based multiprofessional PPC team seems to be the favoured model of care [13]. There is some evidence suggesting that PPC programmes providing specialised services may reduce healthcare resource utilisation by reducing hospital admissions, shortening hospital stays, and lessening aggressive care to prolong life [30]. Additionally, provision of hospital-based specialised PPC may shift and extend the care setting beyond the hospital [30]. In order to provide good quality EOL care a high level of expertise with a good understanding of the different illness trajectories, and efficient collaboration across a variety of paediatric subspecialties is required. Outcome measurement has to be introduced into practice and prospective studies are needed to evaluate meaningful family-oriented clinical outcomes, quality of care, and the impact of specialised PPC to advance clinical practice and research in the field [30, 31].

## Conclusions

Swiss paediatric patients, similar to what is reported from other countries, experience high-intensity EOL care with invasive interventions, high medication counts and high symptom prevalence. Transitions between the inpatient and the home care setting were experienced by most patients outside the neonatology group. Professional home care was only utilised by half of the patients/families. The provision of specialised palliative and EOL care services, including the capacity of community healthcare services were limited. There is therefore a need for PPC services to be expanded to meet the specific needs of seriously ill children and their families in Switzerland.

## Abbreviations

CCC: Complex chronic conditions
CPR: Cardiopulmonary resuscitation
DNR: Do not resuscitate
EOL: End-of-life
ICD: International Statistical Classification of Diseases and Related Health Problems
ICU: Intensive care unit
IQR: Interquartile range
PELICAN: Paediatric End-of-LIfe CAre Needs
PPC: Paediatric palliative care
